# Factors Informing the Return of Adopted Dogs and Cats to an Animal Shelter

**DOI:** 10.3390/ani10091573

**Published:** 2020-09-03

**Authors:** Sloane M. Hawes, Josephine M. Kerrigan, Tess Hupe, Kevin N. Morris

**Affiliations:** Institute for Human-Animal Connection, Graduate School of Social Work, University of Denver, Denver, CO 80208, USA; sloane.hawes@du.edu (S.M.H.); josephine.kerrigan@aol.com (J.M.K.); tess.hupe@du.edu (T.H.)

**Keywords:** companion animals, cat, dog, shelter, outcomes, euthanasia, returned adoption, length of stay

## Abstract

**Simple Summary:**

This study examined the reasons cats and dogs are returned following adoption at one shelter in Austin, TX, USA. The study found that dogs were most likely to be returned for behavioral issues while cats were most likely to be returned for personal reasons. The length of ownership before the pet was returned to the shelter varied substantially. These data can be used in future discussions of how to develop pet retention programs that address the factors informing returned adoptions.

**Abstract:**

Although the adoption rate of dogs and cats from animal shelters has increased, a proportion of animals are returned to the shelter after they are adopted. The purpose of this study was to assess the factors informing the return of 102 dogs to an animal shelter over a four-month period, and the return of 72 cats to an animal shelter over a three-month period. Descriptive statistics revealed dogs are most commonly returned for behavior issues related to aggression (38.2%), and cats are most commonly returned due to the adopter’s personal reasons (56.9%). The results also indicated that more than half of the dogs (51.0%) and cats (57.0%) returned in this study were owned for more than 60 days. Further research is needed to compare the effectiveness of different pet retention programs in addressing the factors that inform returned adoptions.

## 1. Introduction

In the US, the number of companion animals returned to animal shelters within the first six months of adoption is estimated to be anywhere from 7% to 20% [1,2,3,4,5]. Although the number of dogs and cats euthanized in shelters has decreased approximately ten-fold from an estimated 64 per 1000 capita in the 1970 s to a currently estimated 5.6 per 1000 capita [6,7], if animals are returned or relinquished for reasons related to illness, injury, old age, or behavior issues that threaten safety they may be at an increased risk of euthanasia [8,9,10]. Addressing the issue of pet retention is important due to the positive role pets play in communities [11,12]. Numerous studies have shown that companion animals can contribute to both human physical [13,14,15,16,17] and psychosocial health [18,19,20,21,22]. On a larger scale, companion animals can help enhance community cohesion and social capital by facilitating social interaction, friendships, support networks, and civic engagement [23,24], and pet ownership has been shown to positively contribute to local economies [25,26,27]. Research by the American Humane Association (AHA) identified pet homelessness as a complex issue that is impacted by overpopulation of unhoused pets as well as inadequate and misdirected resources to care for companion animals in communities [4]. Therefore, understanding the full scope of issues related to the reasons for the return of adopted dogs and cats can inform the development of pet-support programs that can help animal shelter and rescue organizations more effectively address pet retention issues. 

Most of the research addressing the issue of adopted animals being returned to shelters has focused on dogs [9,28,29,30]. In a study conducted in Northern Ireland [28], 556 people completed a survey within the first month of being a pet owner about their experience of dog acquisition. Approximately 12% of respondents stated they no longer had the adopted dog in their home. Out of those who no longer owned their dog, over half (56%) indicated that they had returned their dog to a shelter within the first month. Almost 90% of the participants who returned their dog to a shelter revealed they did so because of behavior issues, and the most common behavior issue that resulted in a return to the shelter was aggression toward humans [28]. Another study looking at dogs who were returned after adoption from a public shelter in Italy found that over half of the participants (54%) returned their dogs due to a behavior issue that included vocalization, hyperactivity, destruction, escaping, disobedience, and aggression [29]. The remainder of the participants who provided a reason for return identified personal problems related to human health, their inability to take care of the animal, or housing issues. This study also found no correlation between length of stay at the animal shelter and the reason pets were returned. Marston, et al. [9] analyzed the reasons for post-adoption returns of shelter dogs in Australia and found that out of 318 dogs returned to the shelter, approximately 26% were returned for owner-related reasons (e.g., moving, inappropriate selection, etc.), 22% for animal-related problems (e.g., size, health, etc.), 22% for behavioral issues, 13% because of complications between the new dog and another resident pet, and 17% did not record a reason. A follow-up study collected similar results with owner factors, behavior concerns, and dog factors comprising the most frequent reasons for return [30].

While research investigating the return of cats to shelters is less common, Shore [31] interviewed 78 individuals after they returned either a dog or cat to a shelter, which included the return of 82 dogs and 18 cats. Although the findings were not categorized by species, the new cat or dog not getting along with another pet in the home and problems between the pet and a child were the most frequently reported reasons for return (28.0%). One third of the returned adoptions in the study were due to other behavioral issues, such as inappropriate elimination, escaping, aggression, shyness, high activity, and destruction, while other reasons, including allergies, illness of the animal, not enough time, the family moving to a new home, and unknown accounted for the rest [31]. In one relatively large and geographically representative study of the factors informing pet retention, data were collected from 572 individuals that adopted dogs or cats from six different shelters located in three US cities [4]. It was found that just over 10% of the adopted pets were no longer in the home within the first six months after adoption, with approximately 40% returned to a shelter. Younger people (ages 25–34) and those who received advice and support from loved ones or professionals had higher rates of pet retention. However, factors such as doing research on pet ownership prior to adoption and having previous pet ownership experience were found to have no impact on the rate of pet retention. Pet owners who had concerns related to cost, time, health, or animal behavior were more likely to return their pet to a shelter. Although it appeared as if pets who had remained in the home were more likely to have seen a veterinarian than those not retained, the authors noted that no conclusions could be drawn about how seeing a veterinarian influenced pet retention due to the possibility that some owners were waiting to take their new pet to see a veterinarian until they were positive they were going to keep the dog or cat. The study concluded that more preventative measures to help set expectations for pet owners at the time of adoption and to connect them with supportive resources may be more effective pet retention strategies than relying on other post-adoption programming options [4].

This study examines the issue of returned adoptions of both dogs and cats at a non-profit animal shelter in Austin, Texas, USA and discusses how these factors could be addressed through pet retention programs. Data were collected on the length of pet ownership, reasons for return, the most recent outcome for the animal since they were returned, and the owner’s awareness and use of pet retention services offered by APA. This study further addresses potential factors that inform returned adoptions and considers implications for more effectively addressing issues of relinquishment and returns.

## 2. Materials and Methods 

### 2.1. Data Collection

The private companion animal shelter included in this study focuses on serving animals who are at risk of euthanasia or long lengths of stay in other shelters and rescue organizations in the state of Texas, USA. Animals primarily enter the care of the shelter through their transfer partnerships with other animal shelters, owner/guardian surrender, returned adoptions, being born in shelter care, or from other community sources (Table 1). The organization was selected for this study due to its collection of innovative programs that are reported to result in a higher rate of live outcomes for animals that are usually considered difficult to place and its adherence to collecting detailed data that can be used to evaluate program outcomes. The programs offered by the shelter following adoption (see below) attempt to support retention by addressing the medical and behavioral challenges that have typically informed reasons for returned adoption or relinquishment of dogs and cats to shelters. 

The post-adoption support programs that the shelter offers include the a program that helps community members identify alternative solutions to relinquishment, client referral to a low-cost veterinary care organization, a behavior support team, and a free wellness check-up. The shelter’s program to help community members identify alternatives to relinquishment was created in partnership with the local municipal shelter to reduce relinquishment related to circumstances such as changes in housing accommodations, financial difficulty, or other personal issues. This program provides temporary pet retention support through counseling, education, and financial assistance so that pet owners can keep their pet in their home or have access to a foster placement until circumstances are more stable. The low-cost spay/neuter organization that the shelter refers to is a non-profit organization that provides affordable and accessible veterinary care services to pet owners through a network of local clinics. The shelter includes information on this organization as a resource on their website, within their adoption take home paperwork, and through the post-adoption follow-up emails that are sent following adoption by the shelter’s volunteers. The shelter’s behavior support team is a complimentary service for all dogs and cats adopted from the shelter that provides advice for behavioral issues (not including basic obedience) via phone and private sessions. The behavior support team provides unlimited lifetime support for all adopters. All adopters are provided information about the behavior support team available through the shelter at the time of adoption, on the shelter’s website, and through the post-adoption follow-up emails. Within the adoption packet provided to new adopters, there is also a coupon for a free wellness exam and one dose of flea/tick and heartworm preventative. The packet provides information on four locations near the shelter where the coupon is redeemable. Finally, if an animal was diagnosed with a medical condition prior to adoption (e.g., FeLV, Distemper, etc.), the shelter will continue to provide support on a case to case basis (e.g., prescription cost, adopter or medical counseling, etc.).

As a stipulation of their adoption contract, the shelter requires that if an animal who is adopted from their shelter needs to be rehomed, that animal must be returned to the shelter’s custody rather than surrendered to another shelter or rescue organization. A retrospective cohort study was conducted on data collected by the shelter about the animals who were returned using a common database for animal tracking and program evaluation purposes (ShelterLuv, Inc.). Survey data were collected from all individuals returning a dog or cat they had adopted from the shelter administered by a shelter staff member using an electronic tablet. The study sample included any dog returned to the shelter between 3 July 2018 and 22 October 2018 and any cat returned to the shelter between 13 August 2018 and 22 October 2018. In the shelter’s database, intake type is typically classified as a “returned adoption” if the animal had been owned by the adopter for fewer than 30 days and as an “owner/guardian surrender” if the animal had been owned by the adopter for more than 30 days. For the purposes of this study, all dogs and cats who had been adopted from the shelter and then later returned to the shelter’s custody, regardless of the length of time an animal was owned by the adopter, were included in the sample.

Data collected for each animal in the study included species, sex, age group, size group, identified primary breed, breed group (dogs only), length of ownership, and outcome type. When possible, the data were coded into nominal or ordinal variables for the purposes of analysis. The age group for dogs consisted of juvenile, adult, and seniors. The juvenile age group included dogs who were less than 12 months of age. Adult age group included all dogs with an age of 1–7 years. Senior dogs included dogs greater than 7 years old. The age group for cats consisted of juvenile, adult, and seniors. Juvenile cats were any cats less than 6 months of age. Adult cats were 6 months–7 years of age. Senior cats were greater than 7 years old. The size group was determined by the weight of dogs and was coded into groups of small (0–19 lbs), medium (20–59 lbs), and large (60–99 lbs). The weights of cats were coded into the only size group of small (0–19 lbs). Identified primary breed was based upon what was indicated at intake by the shelter’s staff member who conducted the animal’s initial evaluation or what breed was assessed by the original source shelter. Breeds were then grouped according to the National Dog Show categories of: herding (e.g., Australian Shepherd, Collie, etc.), hound (e.g., American English Coonhound, Beagle, etc.), non-sporting (e.g., Bulldog, Boston Terrier, etc.), sporting (e.g., Cocker Spaniel, Golden Retriever, etc.), terrier (e.g., Airedale, Miniature Schnauzer, etc.) toy (e.g., Chihuahua, Havanese, etc.), and working (e.g., Akita, Boxer, etc.) [32]. Length of ownership was calculated from the date of the animal’s adoption date to the date of their return. Length of ownership was coded into ordinal categories that included: fewer than 30 days, between 30–60 days, or greater than 60 days. 

Survey data were collected from all individuals returning a dog or cat. The survey was administered by a shelter staff member using an electronic tablet. Individuals returning an animal to the shelter complete a survey form that requests a detailed explanation of the reason for return. Data are then added to the animal’s ShelterLuv record by the intake counselor to inform future adoption conversations. The reason for return for the animals in the sample was cross-checked using the more detailed written explanation provided in the survey form. If there was a discrepancy between the reason for intake entered by the shelter staff member and the reason for return reported by the individual returning the animal in their return form, the reason cited by the individual returning the animal was used. The categories provided to describe potential reasons for return included: medical needs of the animal (e.g., cancer or parvovirus); cost of medical needs for the animal (e.g., inability to pay for the care needed to treat the animal); inability to afford basic care (e.g., food or housing); the animal’s aggression towards other animals (e.g., chasing dogs or cats); the animal’s aggression towards humans (e.g., biting, growling, or scratching); destructive tendencies (such as digging in the yard or scratching furniture); separation anxiety (e.g., constant barking when away or poorly socialized); an adopter or family member of the adopter with allergies to the animal;, issues with behavior/temperament of resident pet (e.g., fighting or hissing); breed or species restrictions at the adopter’s place of residence; death of owner or family member; medical needs of the adopter; moving; and unrealistic expectations (e.g., those who did not have enough time to give to the animal, the personality of the animal did not fit, a new job event, a new baby, or other personal factors that were unrelated to living situation, health, or cost). 

After their return to the shelter, outcomes for the dogs and cats in the sample included adoption (by an individual other than the individual who returned the animal), return to their owner/guardian (the individual who returned the animal), euthanasia, or still in care. Any animals that were still in the custody of the shelter after 22 October 2018 had an outcome of “still in care”. 

### 2.2. Data Analysis

Data, aggregated by species, were analyzed as descriptive statistics and reported as a percentage of the total population of either dogs or cats.

### 2.3. Sample

The study sample of returned animals consisted of 102 dogs and 72 cats (Table 2, Table 3 and Table 4).

## 3. Results

The most common reason for return of dogs was behavior-related (55.9%), while the most common reason for return of cats was personal reasons (56.9%) (Table 5). Within the behavior-related reasons, dogs were most likely to be returned for aggression toward humans (23.5%) or aggression toward other animals (14.7%), while cats returned for behavior reasons (34.7%) most commonly expressed aggression toward humans (12.5%) or had destructive tendencies (11.1%). Within personal reasons, dogs were most likely to be returned for unrealistic expectations of the adopter (12.7%), while cats were most likely to be returned due to moving (19.4%), medical issues of the adopter (11.1%), or inability to afford basic care (11.1%). Return for companion animal medical reasons accounted for the smallest numbers of returned dogs (8.8%) and cats (5.6%).

Most of the dogs (51%) and cats (57.0%) were returned after being owned for more than 60 days (Table 6). Time to return varied substantially, with over a third of animals returned in fewer than 30 days and over 20% returned after a year.

The shelter offers several pet-support programs to support their adopters in caring for the dogs and cats they adopt. Most adopters are aware of the services (Table 7). However, while a majority of the individuals who returned a dog (89.2%) or cat (65.3%) were aware of the Behavior Team as a resource to them, only a minority of those who returned a dog (43.1%) or a cat (31.9%) actually used the Behavior Team’s services (Table 8).

Within the study period, a majority of the dogs (52.0%) and cats (63.9%) returned had already been adopted into new homes, while most other dogs (44.1%) and cats (33.3%) were still in the care of APA (Table 9).

## 4. Discussion

Although limited to data collected on dogs and cats returned to a single animal shelter during approximately four- and three-month periods, respectively, the findings of the current exploratory study contribute to understanding the factors that inform return of adopted animals.

### 4.1. Factors Informing Reason for Return

A majority (55.9%) of the dogs returned in the present study were returned to the shelter due to behavior issues, which is consistent with prior studies investigating returned adoptions [28,29,31]. Wells and Hepper [28] found that the most common behavior issues identified when returning a dog were aggression toward either animals or humans. These two factors were the most common reason for return of dogs and made up over one third of all dogs returned to the shelter in this study. The third most common reason for return of a dog within this study was unrealistic expectations, which accounted for 12.7% of all dogs returned to APA. These reasons for return of dogs at the shelter in this study are different than the primary reasons a dog is typically admitted to the shelter (Table 1). Most of the general population of dogs were admitted to the shelter due to space issues at the shelter the dog was originally surrendered (36.9%) or medical issues (29.6%).

There was a wider range of reasons cats were returned within this study, but 56.9% of cats were returned for reasons categorized as “personal”. Although Shore [31] did not find moving to be a prominent factor in the reason for returning pets after adoption, in this study, the most common personal reason for return of a cat (19.4%) was moving. The second most frequently cited reason for return of cats to APA was aggression toward humans, which Shore [31] also identified as an issue for the return of pets. Other common factors that contributed to the return of a cat in this study were the inability to afford veterinary care (11.1%), medical needs of the adopter (11.1%), and destructive tendencies of the cat (11.1%). As with dogs, these reasons for return of cats at the shelter in this study are different than the primary reasons a cat is typically admitted to the shelter (Table 1). A majority of the general population of cats were admitted to the shelter due to being a “bottle baby” that needed additional care than could be provided by the shelter the cat was originally surrendered to (28.4%) or medical issues (29.5%).

### 4.2. Length of Ownership

Typically, an animal is considered to be “returned” if they were owned by an adopter for fewer than 30 days. The presumption is that an animal returned within a month of ownership was not an appropriate fit for the family. Shelters typically focus their pet retention efforts within this 30-day period to give the adopters and the animal the best chance of working through any potential challenges they experience once the animal is in their new home. On the other hand, an animal who was adopted but then returned to the shelter after 30 days would typically be classified as an “owner surrender” because the animal was in the home for a more extended period of time and presumably developed issues beyond what was immediately present while the animal was in shelter care. Within this study most dogs (51%) and cats (57%) were owned for over 60 days before they were returned to the shelter. Some of these dogs (22.6%) and cats (29.2%) were returned more than a year after they were adopted. Only about 27% of both dogs and cats were returned within the first two weeks, which contradicts previous studies in which 54% of pets were returned within the first two weeks and only 39% of the participants returned their pets after owning them for more than 60 days [31]. Similarly, another study that found 40% of dogs were returned within the first week [29]. These contradictory results raise questions regarding how pet retention is defined and measured following adoption. In particular, using subjective definitions based on the length of time an animal was in the home to determine whether an animal is classified as a “return” compared to an “owner surrender” may be limiting the understanding of the factors informing the challenges associated with pet retention. 

The shelter in this study offers post-adoption support to adopters regardless of the time that has passed since they adopted an animal. Consequently, the observed length of ownership for animals who were ultimately returned may be an indicator that their post-adoption programs are prolonging the length of ownership before an animal is returned to the shelter with the intention of promoting pet retention. However, more research is needed to understand if extending the length of ownership does, in fact, result in permanent pet retention. The selection criteria for the present study did not lend itself towards answering that particular research question by comparing the number of animals in the post-adoption programs who remained in their homes compared to those that were ultimately returned or relinquished. The results of this study indicate that shelters may want to consider redefining the length of time when an animal is considered to be a “return” compared to “relinquishment” in order to better assess the effectiveness of their adoption processes and post-adoption support. Furthermore, if the goals of post-adoption programs are to support pet retention efforts and decrease rates of return, shelters may also consider extending the length of time new adopters are eligible to access these resources.

### 4.3. Implications for Pet Retention Programs

The shelter in this study offers several post-adoption programs designed to assist adopters with challenges interfering with the human-animal bond or their ability to access the services their pets need. In this study, most of the individuals who returned a dog or a cat were aware of the post-adoption pet-support services that are available through the shelter, but only a portion of the individuals who returned an animal actually used those services. Limited research supports several different approaches to pet retention programs that can address the issue of relinquishment and returned adoptions. A 2014 meta-analyses on pet relinquishment revealed that only 17 studies have addressed interventions for pet relinquishment since 2000 [33]. A common theme of interventions that emerged among the studies included provision of resources for caretakers, increased access to veterinary care, and providing pet owners with advice for handling behavior issues [33]. Most studies focused on using education to address the problem, and some of the studies discussed the ways education may function as a protective and proactive approach to the factors that inform relinquishment. However, more research is needed to identify the most effective interventions for relinquishment and returns, because no studies comparing the effectiveness of one intervention compared to another have been conducted to date. Furthermore, to determine the effectiveness of educational initiatives, more information is needed on the best sources, format, and timing to deliver information as well as how often it should be delivered [33].

The acceptance of preventative education as the best strategy for reducing relinquishment is prevalent in the published literature on this topic [2,29,31,33,34]. However, this approach largely ignores the systemic issues that might have a greater influence on whether an individual is able to keep their pet, regardless of the level of information they have about petkeeping, such as access to pet-support services (e.g., low-cost veterinary care, behavior training, information on basic pet wellness needs) or pet-friendly policies in their community (e.g., non-breed, species, or size restrictive rental policies, co-sheltering options for emergency housing). One study conducted with low income individuals who relinquished their pets due to the cost associated with caring for their pets concluded that there is a need for better marketing and accessibility of low-cost pet care services to help curb relinquishment and returns in these communities [35]. In another study, phone surveys administered to assess the experience of re-homing dogs and cats (e.g., given to a family member, friend, veterinarian, or stranger or returned/relinquished to a shelter) identified the availability and accessibility of affordable services as issues contributing to pet owners’ inability to keep their pets [36]. When asked which free or low-cost services may have helped prevent re-homing of the pet, 40% selected veterinary care, 34% selected behavior training, 33% selected access to pet-friendly housing, 30% selected spay and neuter services, 30% selected pet food, 30% selected temporary pet care/boarding, and 17% selected pet deposits [36]. In the present study, 15.8% of the dogs and 34.7% of the cats were returned for personal reasons that are informed by systemic issues, including inability to afford basic care, moving, and breed- or species-specific rental restrictions. 

Furthermore, there is a prevailing assumption that people of color are less likely to use pet care services when they are offered [37,38]. Many of the studies evaluating how demographic characteristics of pet owners impact the use of veterinary services contain highly biased sampling methods or survey instruments that contain language reflecting degrading cultural assumptions about lower socioeconomic status communities or individuals of color [37,39,40,41]. Yet, a recent study demonstrated that when structural barriers (e.g., transportation and affordability) are removed for these populations, race and ethnicity does not predict willingness to use veterinary services [42]. In addition, even further, Latino/a and Black pet owners are more likely to accept subsidized pet sterilization services on first contact than White individuals. Additional research found that cost of veterinary care and the language barriers that make services inaccessible, not race or ethnicity, are barriers to individuals of color seeking companion animal services [43]. More research is needed to further explore how interventions that confront systemic animal welfare issues can impact the rate or returned adoptions and pet relinquishment.

This study identified behavior issues as the most common reason for returning a dog. Multiple studies have found that participating in behavior training courses decreases adopted dog behavior issues [44,45,46,47]. Yet, each of these studies consisted of voluntary participants completing self-report questionnaires about their dog’s behavior, and the severity of the behavior issues these dogs experienced was not established. It appears the behavior support program provided by APA was not always used by individuals who returned a dog. Although 55.9% of the dogs in this study were returned for reasons related to behavior, only 43.1% of individuals returning a dog had contacted the Behavior Team prior to returning their pet. Only 17.6% sought services from the Behavior Team more than once. On the other hand, 34.7% of the cats were returned for reasons related to behavior and 32% stated they had contact with the Behavior Team, with 7% of the participants indicating they contacted the Behavior Team more than once. Coren [48] found that only 25% of dog owners in the US engage in any kind of formal training services, which is consistent with the lower levels of behavior program use observed in this study. However the finding that most of the individuals who returned a cat for a behavior reason had first contacted the behavior support program indicates that there may be some differences in the use of the behavior program for cats compared to dogs. This lack of contact with support services seen by the individuals who returned a dog is consistent with the trends observed in another study, where only 14% of dog owners and 18% of cat owners contacted the animal shelter prior to re-homing their pet [34]. As with this study, there was no analysis as to why pet owners had hesitancy in contacting the shelter or other support services for help before giving up their animal. Future studies should consider collecting data on why certain services are not used by individuals returning their animal. More research is needed to identify the most effective strategies for engaging pet owners in behavior support programs and other pet retention programs that might decrease the rates of relinquishment and returned adoptions.

### 4.4. Impacts on Shelter Outcomes

In this study, only 1% of dogs and 2.8% of cats were euthanized after being returned to the shelter. Another 52% of dogs and 63.9% of cats were adopted, while 44.1% of dogs and 33.3% of cats are still in the care of the shelter. These outcomes are largely a reflection of the shelter’s operational model, where euthanasia is only used in cases of severe medical or behavior issues. Patronek and Crowe [49] measured that dogs returned within 30 days to an organization in Tucson, AZ, USA with a similar operational model as the shelter in the current study had a live release rate of about 97%, while those who were categorized as owner surrenders had a live release rate of close to 88%. However, few studies have investigated the outcomes for returned adoptions in other shelters. Previous findings indicate that 40–50% of dogs returned to shelters are typically euthanatized [5,9]. One of the studies found the most common reasons cited for euthanasia of returned animals were related to concerns of animal health/size, escaping behaviors, and separation anxiety [9]. The findings of the current study are consistent with those found in Patronek and Crowe [49] and indicate that it is possible to have high rates of live release for dogs and cats returned to a shelter. However, more research on strategies for reducing the number of animals relinquished or returned are needed to support other shelter organizations who have not had the capacity to obtain similar rates of live outcomes for animals in their care [5,9].

### 4.5. Limitations

One limitation of the current study is that data presented were specific to one shelter in Austin, TX, USA over a limited time frame of four and three months for dogs and cats, respectively. Both design considerations limit the generalizability of these findings to other animal shelter or rescue organizations. Another limitation is that the design lacked a comparison group of animals who demonstrated similar medical or behavioral challenges but were not returned to the shelter after being adopted. Without these data, there cannot be a quantitative assessment of which factors are more likely to contribute to returning a pet or analyze the effectiveness of the shelter programs in achieving the goal of pet retention. Future studies should incorporate such a comparison group to allow for additional understanding of factors informing pet retention. 

Another consideration to note is that this study used the primary reason for return cited by the individual who returned the animal (determined by reviewing the short answer responses provided in the survey), rather than the intake type recorded by the shelter staff member conducting the intake. This strategy was employed because there were several incidences where a discrepancy existed between the reason for return provided by the individual returning the pet and the intake type that was ultimately entered by the shelter staff member. These discrepancies happened most often when an individual returning an animal reported a behavior-related issue as the reason for return, and then “unrealistic expectations” was entered by shelter staff as the reason for intake instead. Previous research has indicated that the reasons for relinquishment indicated by an owner in more in-depth interviews are often more complex than are reported at the time of intake to a shelter [50]. Since not all shelters record the information directly provided by the individual returning an animal, it is possible that the findings of this study may be inconsistent with other studies that use the reason for return that is entered by a shelter staff member at the time of intake.

## 5. Conclusions

This study presents a summary of the most prevalent reasons for returning a cat or dog adopted from a shelter, in Austin, TX, USA, with the majority of dogs being returned for behavioral issues and the majority of cats for reasons categorized as personal. The findings suggest that pre- and post-adoption services should focus on addressing the behavioral and systemic issues (e.g., access to affordable behavior or medical care) that create substantial barriers for pet retention. Furthermore, the findings illustrate how extending the definition of returned adoptions past 30 days post-adoption can allow for a more comprehensive assessment of pet retention challenges following adoption.

## Figures and Tables

**Table 1:** Reasons for intake of all dogs (*n* = 1604) and all cats (*n* = 1292) who came into the care of the shelter during the study period (3 July 2018 to 22 October 2018 for dogs and 13 August 2018 to 22 October 2018 for cats).

Reason for Intake	Dogs (*n* = 1604)	Percentage of Dog Sample	Cats (*n* = 1292)	Percentage of Cat Sample
Medical		29.6%		29.5%
Medical needs of animal	249	15.5%	349	27.0%
Medical costs	2	0.1%	0	0.0%
Urgent medical	38	2.4%	14	1.1%
Parvovirus	186	11.6%	0	0.0%
Panleukopenia	0	0.0%	18	1.4%
Behavioral		9.2%		5.9%
Aggressive toward animals	27	1.7%	3	0.2%
Aggressive toward humans	37	2.3%	3	0.2%
Destructive tendencies	12	0.8%	5	0.4%
Separation anxiety	7	0.4%	1	0.08%
Behavior/temperament	7	0.4%	5	0.4%
General behavioral support needed	57	3.6%	59	4.6%
Personal		8.6%		8.4%
Cannot afford basic care	22	1.4%	10	0.8%
Unrealistic expectations	61	3.8%	24	1.9%
Moving	30	1.9%	31	2.4%
Unwanted offspring of pet	10	0.6%	0	0.0%
Medical needs of pet owner	6	0.4%	14	1.1%
Allergies	2	0.1%	8	0.6%
Behavior/temperament of resident pet	4	0.2%	18	1.4%
Death of owner/family member	4	0.2%	2	0.2%
Other		52.6%		56.2%
Born in shelter care	105	6.5%	29	2.2%
Stray with shelter’s microchip	14	0.9%	10	0.8%
Stray	14	0.9%	20	1.5%
Breed or species restrictions	3	0.2%	0	0.0%
Wellness	1	0.06%	0	0.0%
Bottle baby	28	1.7%	367	28.4%
Pregnant/nursing	86	5.4%	118	9.1%
Shelter space	592	36.9%	184	14.2%

**Table 2:** Description of the sample of dogs (*n* = 102) and cats (*n* = 72) by number of animals and percentage of the samples.

Descriptive	Dogs (*n* = 102)	Percentage of Dog Sample	Cats (*n* = 72)	Percentage of Cat Sample
Sex				
Female	47	46.1%	38	52.8%
Male	55	53.9%	33	45.8%
Unknown	0	0.0%	1	1.4%
Age Group				
Juvenile (<1 year old)	19	18.6%	21	29.2%
Adult (1–7 years old)	69	67.7%	45	62.5%
Senior (>7 years old)	14	13.7%	6	8.3%
Size Group				
Small (0–19 lbs.)	16	15.7%	72	100.0%
Medium (20–59 lbs.)	60	58.8%	0	0.0%
Large (60–99 lbs.)	26	25.5%	0	0.0%

**Table 3:** Dog breed group by number of animals and percentage of the sample (*n* = 102).

Breed Group	Dogs (*n* = 102)	Percentage of Dog Sample
Herding	22	21.6%
Hound	6	5.9%
Non-Sporting	4	3.9%
Sporting	18	17.6%
Terrier	28	27.5%
Toy	5	4.9%
Working	19	18.6%

**Table 4:** Cat breed group by number of animals and percentage of the sample (*n* = 72).

Breed Group	Cats (*n* = 72)	Percentage of Cat Sample
Domestic Longhair	5	6.9%
Domestic Medium Hair	2	2.8%
Domestic Shorthair	58	80.5%
Japanese Bobtail	1	1.4%
Manx	1	1.4%
Russian Blue	2	2.8%
Siamese	3	4.2%

**Table 5:** Reasons for return by number of animals and percentage of the sample for cats and dogs who were returned to the shelter during the study period (3 July 2018 to 22 October 2018 for dogs and 13 August 2018 to 22 October 2018 for cats).

Reason for Return	Dogs (*n* = 102)	Percentage of Dog Sample	Cats (*n* = 72)	Percentage of Cat Sample
Medical		8.8%		5.6%
Medical needs of animal	6	5.9%	3	4.2%
Medical costs	3	2.9%	1	1.4%
Behavioral		55.9%		34.7%
Aggressive toward animals	15	14.7%	7	9.7%
Aggressive toward humans	24	23.5%	9	12.5%
Destructive tendencies	11	10.8%	8	11.1%
Separation anxiety	7	6.9%	1	1.4%
Personal		31.4%		56.9%
Allergies	3	2.9%	6	8.3%
Behavior/temperament of resident pet	3	2.9%	3	4.2%
Inability to afford basic care	2	2.1%	8	11.1%
Death of owner or family member	0	0.0%	1	1.4%
Medical needs of adopter	4	3.9%	8	11.1%
Moving	7	6.9%	14	19.4%
Unrealistic expectations ^1^	13	12.7%	1	1.4%
Other		3.9%		2.8%
Breed or species restrictions	4	3.9%	2	2.8%

^1^ There are a small number of discrepancies in the number of animals within each of the reasons for return categories in Table 5 and the number of animals within each reason for intake reported for the total population of animals admitted into the shelter’s care in Table 1 (primarily where animals entered under the category of “aggressive towards animals” or “aggressive towards humans” as a reason for return were coded as “unrealistic expectations” as an intake type). If there was a discrepancy between the reason for intake entered by the shelter staff member and the reason for return reported by the owner in their return form, the reason cited by the owner was used in Table 5, whereas the reason for intake, as assessed by the shelter staff member were used for Table 1.

**Table 6:** Length the dog or cat was owned prior to return by number of animals and the percentage of the sample.

Length of Ownership	Dogs (*n* = 102)	Percentage of Dog Sample	Cats (*n* = 72)	Percentage of Cat Sample
<30 Days		42.2%		34.7%
0–2 Weeks	28	27.5%	20	27.8%
2–4 Weeks	15	14.7%	5	6.9%
30–60 Days		6.8%		8.3%
4–6 Weeks	4	3.9%	5	6.9%
6–8 Weeks	3	2.9%	1	1.4%
>60 Days		51.0%		57.0%
2–4 Months	8	7.8%	8	11.1%
4–6 Months	6	5.9%	5	6.9%
6–8 Months	10	9.8%	4	5.6%
8–10 Months	4	3.9%	1	1.4%
10–12 Months	1	1.0%	2	2.8%
>1 Year	23	22.6%	21	29.2%

**Table 7:** Awareness of pet-support services provided by the shelter.

Did you Have Awareness of the Following Services?	Dogs (*n* = 102)	Percentage of Dog Sample	Cats (*n* = 72)	Percentage of Cat Sample
Behavior Team				
Yes	91	89.2%	47	65.3%
No	5	4.9%	15	20.8%
No Response	6	5.9%	10	13.9%
Low-Cost Veterinary Care				
Yes	68	66.7%	42	58.3%
No	26	25.5%	20	27.8%
No Response	8	7.8%	10	13.9%
Free Wellness Check-Up				
Yes	80	78.4%	45	62.5%
No	14	13.7%	17	23.6%
No Response	8	7.9%	10	13.9%
Alternatives to Surrender Program				
Yes	75	73.6%	43	59.7%
No	18	17.6%	20	27.8%
No Response	9	8.8%	9	12.5%

**Table 8:** Use of the Behavior Team resource.

Did you Have Contact with the Behavior Team?	Dogs (n = 102)	Percentage of Dog Sample	Cats (n = 72)	Percentage of Cat Sample
No response	6	5.9%	7	9.7%
No	52	51.0%	42	58.3%
Yes, once or twice	26	25.5%	18	25.0%
Yes, multiple times	18	17.6%	5	7.0%

**Table 9:** Outcome for animals returned by number of animals and percentage of sample.

Outcome	Dogs (*n* = 102)	Percentage of Dog Sample	Cats (*n* = 72)	Percentage of Cat Sample
Adopted	53	52.0%	46	63.9%
Euthanized	1	1.0%	2	2.8%
Returned to owner/guardian	3	2.9%	0	0.0%
Still in care	45	44.1%	24	33.3%
